# Effects of Different Individuals and Verbal Tones on Neural Networks in the Brain of Children with Cerebral Palsy

**DOI:** 10.3390/brainsci15040397

**Published:** 2025-04-15

**Authors:** Ryosuke Yamauchi, Hiroki Ito, Ken Kitai, Kohei Okuyama, Osamu Katayama, Kiichiro Morita, Shin Murata, Takayuki Kodama

**Affiliations:** 1 The Graduate School of Health Science, Kyoto Tachibana University, Kyoto 607-8175, Japan; h901524007@st.tachibana-u.ac.jp (H.I.); h901523004@st.tachibana-u.ac.jp (K.K.); h901523002@st.tachibana-u.ac.jp (K.O.); katayama.o@ncgg.go.jp (O.K.); murata-s@tachibana-u.ac.jp (S.M.); kodama-t@tachibana-u.ac.jp (T.K.); 2 Otemae Rehabilitation Center with Physical Disabilities, Osaka Red Cross Hospital, Osaka 543-8555, Japan; 3 National Center for Geriatrics and Gerontology, Center for Gerontology and Social Science, Obu 474-8511, Japan; 4 Cognitive and Molecular Research Institute of Brain Diseases, Kurume University, Fukuoka 830-0011, Japan; kiichiro@kurume-u.ac.jp

**Keywords:** motivation, rehabilitation, electroencephalogram, cerebral palsy, communication, emotion

## Abstract

**Background/Objectives**: Motivation is a key factor for improving motor function and cognitive control in patients. Motivation for rehabilitation is influenced by the relationship between the therapist and patient, wherein appropriate voice encouragement is necessary to increase motivation. Therefore, we examined the differences between mothers and other individuals, such as physical therapists (PTs), in their verbal interactions with children with cerebral palsy who have poor communication abilities, as well as the neurological and physiological effects of variations in the tone of their speech. **Methods**: The three participants were children with cerebral palsy (Participant A: boy, 3 years; Participant B: girl, 7 years; Participant C: girl, 9 years). Participants’ mothers and the assigned PTs were asked to speak under three conditions. During this, the brain activity of the participants was measured using a 19-channel electroencephalogram. The results were further analyzed using Independent Component Analysis frequency analysis with exact Low-Resolution Brain Electromagnetic Tomography, allowing for the identification and visualization of neural activity in three-dimensional brain functional networks. **Results**: The results of the ICA frequency analysis for each participant revealed distinct patterns of brain activity in response to verbal encouragement from the mother and PT, with differences observed across the theta, alpha, and beta frequency bands. **Conclusions**: Our study suggests that the children were attentive to their mothers’ inquiries and focused on their internal experiences. Furthermore, it was indicated that when addressed by the PT, the participants found it easier to grasp the meanings and intentions of the words.

## 1. Introduction

Motivation during the performance of physiotherapy tasks has been identified as a key factor for improving motor function and cognitive control in patients [1,2,3]. Motivation is a personal characteristic that facilitates the achievement and maintenance of a goal. It is clear that patients’ motivation towards rehabilitation has a significant impact on the therapeutic effectiveness, especially in pediatric rehabilitation [4]. In child development, the active participation and continuous commitment of patients to rehabilitation are indispensable for promoting functional recovery and social adaptation. Additionally, it has been reported that motivation tends to decline over time, and factors such as fatigue, external environment, and reward misalignment are involved in this decrease in motivation [5,6]. Therefore, it is especially important to increase and maintain the motivation of children for pediatric rehabilitation by using preferred tasks, rewards [7], building trust relationships with others, and increasing self-esteem [8,9]. It has been reported that involving family members in the rehabilitation process maximizes the effectiveness of interventions, highlighting that the relationship between the child, family, and therapist is essential [10]. Moreover, the individual–environment interaction is imperative in child development, and the acquisition of motor skills enhances exploratory behaviors and influences perceptual, cognitive, and language development [11]. Therefore, children with cerebral palsy, who have severe motor and intellectual disability (CP-SMID), have impaired mobility owing to motor disabilities. This limits spontaneous exploration, which consequently induces severe intellectual developmental delays, decreased interest in objects and people, and decreased motivation to move and exercise. Therefore, we believe that it is essential to motivate children to move, exercise, and develop an interest in objects.

Motivation for rehabilitation is influenced by the relationship between the therapist and patient [8], wherein appropriate voice encouragement is necessary to increase motivation [12]. Changes in motivation are reported to be associated with changes in emotions. Therefore, it is essential to promote emotional changes in children to enhance their motivation. As children with CP-SMID have severe mental and physical disabilities that frequently include communication disorders, it is difficult to objectively assess how these patients perceive the voices of others, such as their parents and therapists, and ascertain the effect that they have on their motivation. Therefore, it is difficult to objectively evaluate and share children’s emotional changes with others. Kangaroo care, wherein a contact stimulus is given to a neonate who has not yet acquired communication abilities, has been shown to elicit similar physiological responses toward both the mother and father, as measured by heart rate variability (HRV) and apnea/periodicity measures [13]. Additionally, in the field of pediatric rehabilitation, it is essential to collaborate not only with the child who is the patient but also with their family during interventions [14]. It has also been reported that involving the family in rehabilitation enhances the effectiveness of interventions [15]. A study of infants’ sensitivity to language stimuli revealed that infants displayed more sensitivity in their native language rather than in a second/foreign language [16]. These finding has shown that even in infants who have not yet acquired language skills, differences in the individuals providing stimuli and the content of those stimuli can affect emotional and cognitive functions. While autonomic nervous responses, such as heart rate and other physiological measures, have been recognized, there is a lack of research quantifying neural activity and brain networks in children at later stages of communication development. Furthermore, little is known about how differences in vocal tones from various individuals influence these neural networks, enhance emotions, and consequently impact motivation. In light of these considerations, we believe that quantitatively assessing neural activity in the brain using an electroencephalogram (EEG), which has excellent temporal resolution, is essential for clarifying the mental elements necessary for forming a foundation for communication skills in rehabilitation.

We hypothesized that the elements of an emotion-based approach, such as verbal encouragement directed at children in the language development stage (i.e., the mental effects of language), may influence the effectiveness of pediatric rehabilitation. This study aimed to examine how differences in different vocal tones from mothers and therapists affect the neural networks of children with poor communication skills, specifically those with cerebral palsy, using an electroencephalogram (EEG) to clarify the necessary mental elements for forming a foundation for communication skills in rehabilitation.

## 2. Materials and Methods

The present study is designed as an observational study, utilizing a cross-sectional research approach.

### 2.1. Participants

The inclusion criteria for the subjects in this study were individuals diagnosed with CP-SMIDs who were under 12 years old and did not have accompanying hearing impairments, treated at the authors’ affiliated hospital. The exclusion criteria included the following:

1. Severe body movements that could interfere with EEG recording;
2. Hearing impairment that could affect the perception of verbal stimuli;
3. Medical conditions contraindicating the use of EEG equipment.

These criteria were chosen to ensure the quality and reliability of the EEG data collected.

### 2.2. Ethical Considerations

The study was conducted in accordance with the Declaration of Helsinki and approved by Kyoto Tachibana University Research Ethics Committee, (approval number: 22–25; date of approval: 19 August 2022). Informed consent was obtained from all subjects involved in the study. Written informed consent to participate in this research was obtained from the parents/legal guardians of all participants, in accordance with institutional standards.

### 2.3. Stimuli

Speaking directed at the pediatric participants was provided by their mothers and the children’s physical therapists (PTs). The content included the following:

- 20 sentences of speaking based on asking;
- 20 sentences based on informing;
- 40 sentences based on a mixed approach that combined asking and informing.

Although the content and character count were identical across conditions, the tone differed:

- Asking sentences were delivered in a friendly manner;
- Informing sentences were delivered in a non-friendly manner;
- The mixed approach incorporated tones appropriate for both asking and informing.

**Figure 1:**
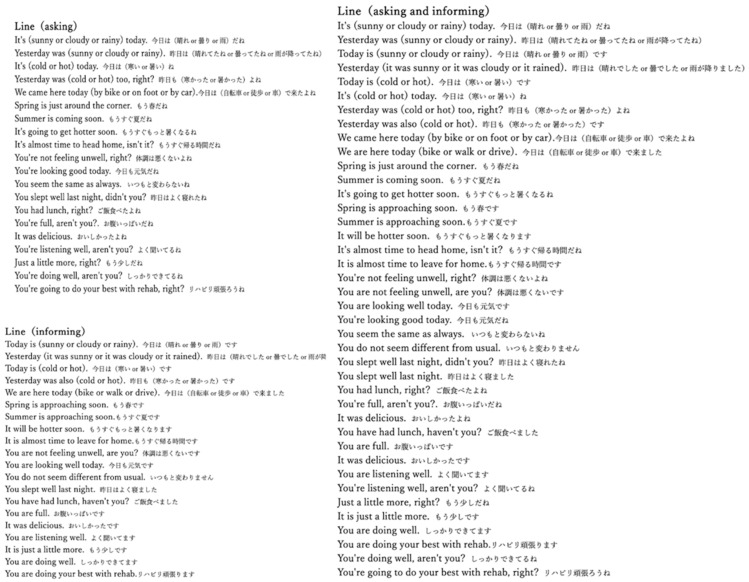
Dialogue examples for different conditions [Asking condition, informing condition, and mixed condition (asking and informing)]. The verbal prompts were conducted in Japanese with English translations provided.

The sentences were developed in collaboration with speech therapists and child psychologists to ensure age-appropriateness and relevance to the rehabilitation context. Mothers reviewed the sentences before measurement to confirm that the content did not evoke undue joy or stress in the child and that there were no elements that could negatively stimulate the child’s emotions. The verbal prompts used in this study were designed to exclude content that would elicit emotional changes, such as those that would either delight the children or insult them. Instead, the focus was on altering the tone and intonation of the questions and instructions. The dialogue examples for different conditions are shown in Figure 1, with both Japanese originals and English translations provided.

### 2.4. Procedure

**Figure 2:**
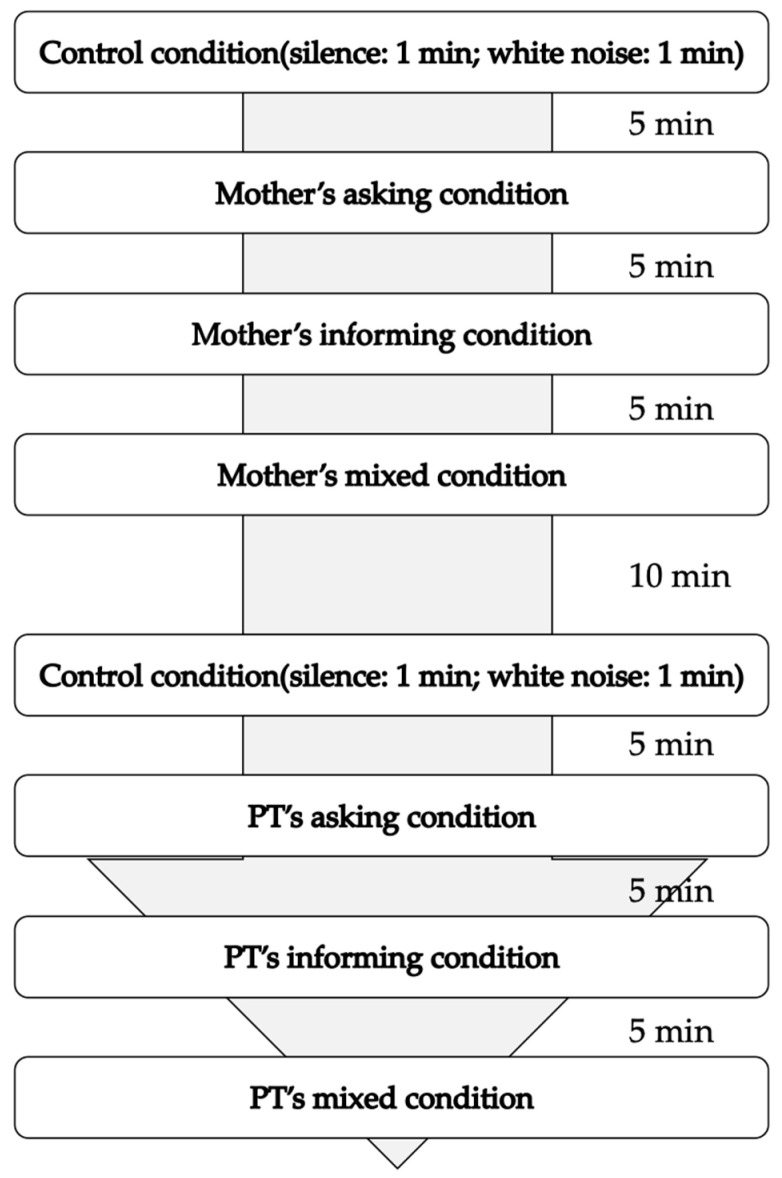
Measurement protocol. Twenty sentences were used for the asking condition, twenty sentences for the informing condition, and forty sentences for the mixed condition. Each sentence was read aloud at three-second intervals, with the asking condition and informing condition lasting approximately three minutes, and the mixed condition lasting about six minutes. Each condition was randomly administered in the first and second measurements.

The measurement protocol is shown in Figure 2. Each session proceeded as follows:

1. Control condition: 1 min EEG recording in silence, followed by 1 min recording with white noise;
2. 5 min break;
3. Mother’s speaking conditions: asking, informing, and mixed, with 5 min breaks between each condition;
4. 10 min break;
5. Repeat of control condition;
6. PT’s speaking conditions: asking, informing, and mixed, with 5 min breaks between each condition.

The measurements were conducted over two days, with the order of the mothers’ and PTs’ speaking reversed between the first and second measurements. The order of asking, informing, and mixed conditions was randomized across sessions to control for order effects. The measurement environment was a quiet, temperature-controlled room in the hospital, designed to minimize external distractions and ensure participant comfort.

### 2.5. EEG Recording and Analysis

Brain neural activity was recorded using the Polymatepro6100 bio-signal recording device (Miyuki Giken Co., Ltd., Tokyo, Japan), based on the international 10–20 system. Measurements were taken from 19 locations: Fp1, Fp2, F3, F4, F7, F8, Fz, C3, C4, Cz, P3, P4, Pz, O1, O2, T3, T4, T5, and T6. Electrode impedances were kept below 5 kΩ. The reference electrodes were placed on the earlobes. The frequency bands analyzed were theta (θ) (4–7 Hz), Alpha (α) (8–13 Hz), and Beta (β) (14–20 Hz). To accurately extract neural activity during verbal encouragement, the muscle activity of the speaker’s suprahyoid muscles was measured as a trigger, allowing synchronization with the child’s EEG measurements. The bandpass filter in the EEG measurement was 0.5–30 Hz, and the sampling rate was 1000 Hz. EEG data analysis was conducted using the Electro Magnetic Source Estimation program (EMSE) (Cortech Solutions, Inc., version 5.5.2). Data for each condition were extracted based on triggers, isolating the waveforms for 2 s after the end of each verbal encouragement stimulus. The data for each condition were then averaged to create the analysis dataset. Independent Component Analysis (ICA) was performed using Matrix Laboratory (MATLAB) (v.R2023b Update 6, MathWorks, 2023). ICA was chosen for its ability to separate EEG data into statistically independent components, allowing for effective artifact removal. Components identified as noise (e.g., eye blinks and muscle artifacts) were visually inspected and removed.

The ICA frequency analysis was conducted using Transposed fICA Networks within exact Low-Resolution Brain Electromagnetic Tomography (eLORETA, version 20160611). This method allows for the three-dimensional visualization of neural activity and the identification of brain functional networks. The fICA networks jointly represent space and frequency interactions, producing three sets of images (θ-wave, α-wave, and β-wave bands). This approach enables the identification of regions working together within each condition and captures coupling between frequencies.

### 2.6. Statistical Analysis

Due to the small sample size, formal statistical tests were not performed. Instead, descriptive analyses were conducted to explore patterns in the EEG data across conditions and participants. The activity values of the regions showing the maximum and minimum activity values were extracted from the activity values calculated by the eLORETA analysis for qualitative comparison.

## 3. Results

### 3.1. Participant Baseline Characteristics

The characteristics of the three participants are summarized in Table 1.

Among the three participants, one was a boy (Participant A, age: 3 years) and two were girls (Participant B, age: 7 years; Participant C, age: 9 years). All participants had CP-SMID, classified as having severe psychosomatic disorders. Moreover, they all faced challenges in verbal communication, vocalizing but struggling to produce meaningful words. The motor level of Participant A was such that he had difficulty turning over and was unable to move voluntarily. The motor level of Participant B was similar to Participant A, as she had difficulty turning over and was unable to move voluntarily. However, the motor level of Participant C had developed to the point where she could crawl a few meters.

### 3.2. EEG Analysis

The results for the main brain activity areas in each frequency band for subjects A, B, and C under each condition are summarized in Table 2.

#### 3.2.1. Participant A

**Figure 3:**
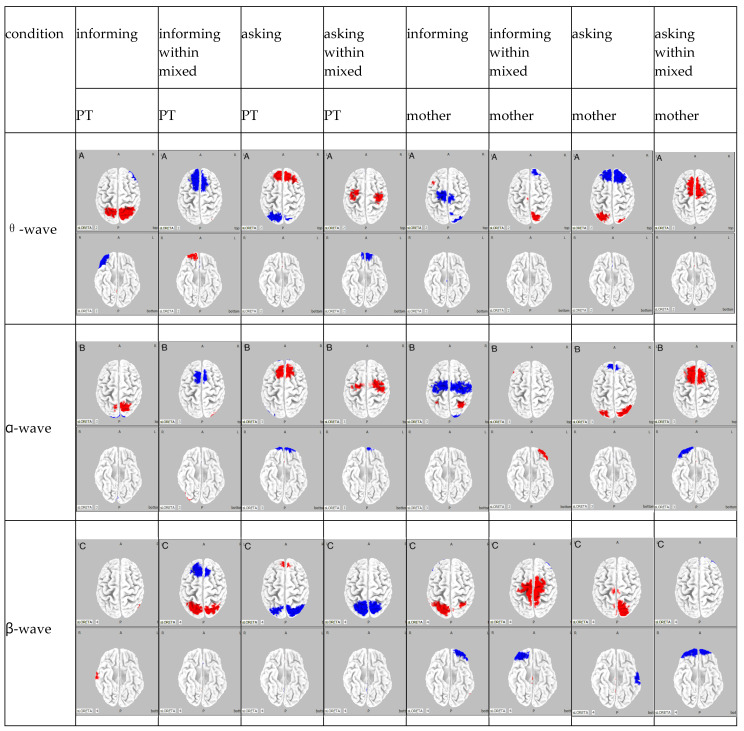
Participant A: The θ wave band for each condition is designated as A, the α wave band as B, and the β wave band as C. The columns are classified by frequency, and the rows are classified by condition. Regions of attenuated activity are shown in blue, whereas regions of amplified activity are shown in red.

The results of the frequency analysis by ICA for Participant A are shown in Figure 3.

The results of the ICA frequency analysis for Participant A indicated the following findings. In the PT informing condition, in the θ wave band, there was an amplification in activity in the bilateral somatosensory association areas and a reduction in activity in the right inferior frontal gyrus; in the α wave band, there was an amplification in activity in the bilateral somatosensory association areas and a reduction in activity in the bilateral visual association area; in the β wave band, there was an amplification in activity in the right superior temporal gyrus and the right middle temporal gyrus. In the mother informing condition, in the θ wave band, there was an amplification in activity in the bilateral frontal eye fields, along with a reduction in activity in the bilateral supplementary motor areas and bilateral visual association areas; in the α wave band, there was an amplification in activity in the bilateral somatosensory association areas and a reduction in activity in the bilateral supplementary motor areas and bilateral visual association areas; in the β wave band, there was an amplification in activity in the bilateral somatosensory association areas and the left superior temporal gyrus, with a reduction in activity in the left frontal pole.

In the PT informing within mixed condition, in the θ wave band, there was an amplification in activity in the right frontal pole and a reduction in activity in the bilateral somatosensory association areas; in the α wave band, there was an amplification in activity in the right visual association area and a reduction in activity in the bilateral supplementary motor areas; and in the β wave band, there was an amplification in activity in the bilateral somatosensory association areas and a reduction in activity in the bilateral frontal eye fields. In the mother informing within mixed condition, in the θ wave band, there was a reduction in activity in the right somatosensory association area and a reduction in activity in the right dorsolateral prefrontal cortex; in the α wave band, there was an amplification in activity in the left orbitofrontal cortex; and in the β wave band, there was an amplification in activity in the bilateral supplementary motor areas and a reduction in activity in the right orbitofrontal cortex.

In the PT asking condition, in the θ wave band, there was an amplification in activity in the bilateral frontal eye fields and a reduction in activity in the bilateral somatosensory association areas; in the α wave band, there was an amplification in activity in the bilateral supplementary motor areas and a reduction in activity in the bilateral frontal poles and the left visual association area; and in the β wave band, there was an amplification in activity in the frontal eye fields and a reduction in activity in the somatosensory association areas. In the mother asking condition, in the θ wave band, there was an amplification in activity in the bilateral somatosensory association areas and a reduction in activity in the frontal eye fields; in the α wave band, there was an amplification in activity in the bilateral visual association areas and a reduction in activity in the frontal eye fields; and in the β wave band, there was an amplification in activity in the somatosensory association areas and a reduction in activity in the inferior temporal gyrus.

In the PT asking within mixed condition, in the θ wave band, there was an amplification in activity in the supplementary motor areas and a reduction in activity in the orbitofrontal cortex; in the α wave band, there was an amplification in activity in the supplementary motor areas and a reduction in activity in the orbitofrontal cortex; and in the β wave band, a reduction in activity was observed in the bilateral somatosensory association areas. In the mother asking within mixed condition, in the θ wave band, there was an amplification in activity in the supplementary motor areas; in the α wave band, there was an amplification in activity in the supplementary motor areas and a reduction in activity in the frontal poles; and in the β wave band, there was a reduction in activity in the orbitofrontal cortex.

Summarizing the results for Participant A, under the PT informing condition, somatosensory information processing increased, whereas visual information processing was suppressed. Conversely, under the mother informing condition, motor preparation was carried out quickly. Additionally, in the PT informing condition, frontal eye field activity increased, leading to controlled attention, whereas under the mother informing condition, somatosensory association area activity increased, directing attention toward bodily sensations. Under the PT informing within mixed condition, somatosensory information processing was suppressed, whereas decision-making increased. Similarly, under the mother informing within mixed condition, somatosensory information processing was suppressed, and motor preparation was carried out quickly. In the PT asking condition, somatosensory information processing was suppressed, whereas goal-directed behavior increased. Conversely, under the mother asking condition, somatosensory information processing increased. Under the PT asking within mixed condition, sensory information processing was suppressed, whereas concentration on attention tasks increased. However, in the mother asking within mixed condition, motor preparation was carried out quickly.

#### 3.2.2. Participant B

**Figure 4:**
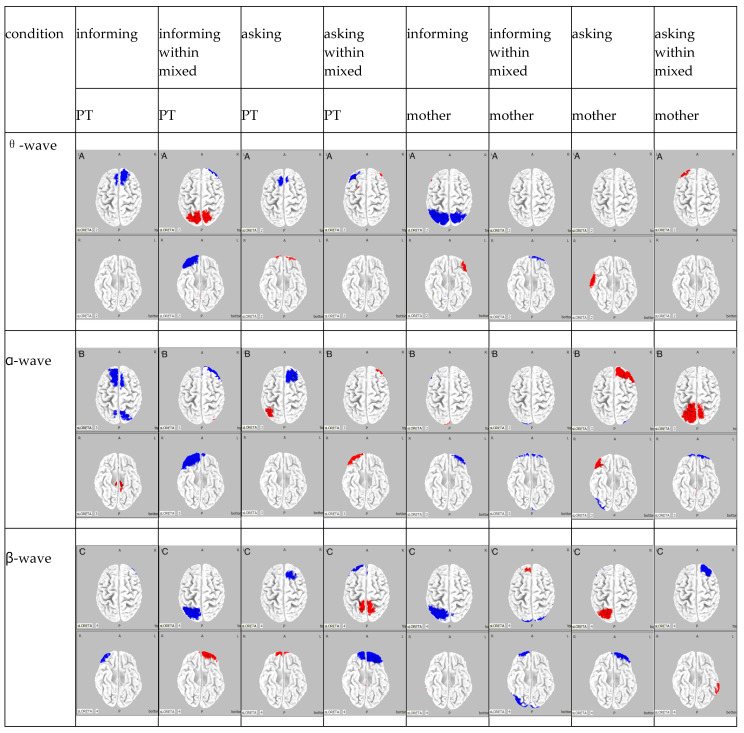
Participant B: The θ wave band for each condition is designated as A, the α wave band as B, and the β wave band as C. The columns are classified by frequency, and the rows are classified by condition. Regions of attenuated activity are shown in blue, whereas regions of amplified activity are shown in red.

The results of the frequency analysis by ICA for Participant B are shown in Figure 4.

The results of the ICA frequency analysis for Participant B indicated the following findings. In the PT informing condition, in the θ wave band, there was a reduction in activity in the dorsolateral prefrontal cortex; in the α wave band, there was an increase in activity in the bilateral dorsal posterior cingulate cortex, along with a reduction in activity in the bilateral somatosensory association areas and the bilateral supplementary motor areas; and in the β wave band, a reduction in activity was observed in the frontal poles. In the mother informing condition, in the θ wave band, there was an amplification in activity in the left inferior frontal gyrus, along with a reduction in activity in the bilateral somatosensory association areas; in the α wave band, there was an amplification in activity in the left visual association area and a reduction in activity in the left orbitofrontal cortex; and in the β wave band, there was an amplification in activity in the right middle temporal gyrus and a reduction in activity in the left somatosensory association area.

In the PT informing within mixed condition, in the θ wave band, there was an amplification in activity in the bilateral somatosensory association areas and a reduction in activity in the orbitofrontal cortex; in the α wave band, there was an amplification in activity in the right frontal pole and a reduction in activity in the visual association areas; and in the β wave band, there was an amplification in activity in the frontal poles and a reduction in activity in the somatosensory association areas. In the mother informing within mixed conditio, in the θ wave band, there was a reduction in activity in the left frontal pole and the right fusiform gyrus; in the α wave band, there was a reduction in activity in the bilateral orbitofrontal cortices and the bilateral visual association areas; and in the β wave band, there was an increase in activity in the left frontal eye fields and a reduction in activity in the right frontal pole and the bilateral visual association areas.

In the PT asking condition, in the θ wave band, there was an amplification in activity in the bilateral orbitofrontal cortices and a reduction in activity in the bilateral supplementary motor areas; in the α wave band, there was an amplification in activity in the somatosensory association areas and a reduction in the supplementary motor areas; and in the β wave band, there was an amplification in activity in the bilateral frontal poles and a reduction in the supplementary motor areas. In the mother asking condition, in the θ wave band, there was an amplification in activity in the right middle temporal gyrus; in the α wave band, there was an amplification in activity in the right dorsolateral prefrontal cortex, along with a reduction in activity in the right visual association areas; and in the β wave band, there was an amplification in activity in the left somatosensory association area and a reduction in activity in the left frontal pole.

In the PT asking within mixed condition, in the θ wave band, there was an amplification in activity in the frontal pole and a reduction in activity in the dorsolateral prefrontal cortex; in the α wave band, there was an amplification in activity in the right frontal pole; and in the β wave band, there was an amplification in activity in the somatosensory association areas and a reduction in activity in the frontal pole. In the mother asking within mixed condition, in the θ wave band, there was an amplification in activity in the left frontal pole; in the α wave band, there was an amplification in activity in the bilateral somatosensory areas and a reduction in activity in the bilateral frontal poles; and in the β wave band, there was an amplification in activity in the bilateral middle temporal gyri and a reduction in activity in the right frontal pole.

Summarizing the results for Participant B, under the PT informing condition, activity in the somatosensory association area was suppressed, whereas activity in the dorsal posterior cingulate cortex increased, indicating that sensory information processing was carried out selectively. Conversely, in the mother informing condition, activity increased in the visual association area and decreased in the orbitofrontal cortex, suggesting that visual information processing was prioritized. Additionally, in the PT informing condition, activity in the frontal pole increased, whereas activity in the supplementary motor area decreased, indicating that cognitive function was enhanced while motor preparation was suppressed. In the mother informing condition, activity increased in the middle temporal gyrus and the somatosensory association area, suggesting that attention was focused on language comprehension and bodily sensations. Under the PT informing within mixed condition, we observed a decrease in activity in the somatosensory association areas and an increase in activity in the frontal pole. This indicates suppression in attention to somatosensory information, whereas highlights an enhancement in decision-making. In the mother informing within mixed condition, simultaneous decrease in activity in the right frontal pole and an increase in activity in the left orbitofrontal cortex were observed. This indicates that higher cognitive processing was suppressed while decision-making was enhanced. Under the PT asked condition, activity in the frontal pole increased, whereas activity in the supplementary motor area decreased, indicating enhanced cognitive functions and reduced attention to movement. In the mother asking condition, activity increased in the somatosensory association area and decreased in the frontal pole, suggesting that sensory processing was enhanced while cognitive processing was diminished. Under the PT asking within mixed condition, activity increased in the somatosensory association area and decreased in the frontal pole, indicating that sensory processing was enhanced while cognitive processing was suppressed. However, in the mother asking within mixed condition, activity increased in the middle temporal gyri and decreased in the frontal pole, suggesting that language comprehension was enhanced while cognitive processing was suppressed.

#### 3.2.3. Participant C

**Figure 5:**
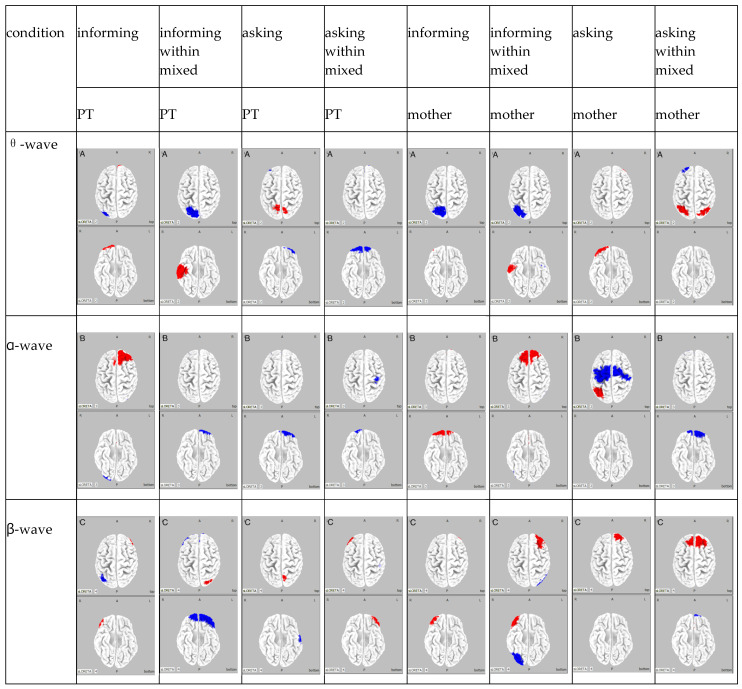
Participant C: The θ wave band for each condition is designated as A, the α wave band as B, and the β wave band as C. The columns are classified by frequency, and the rows are classified by condition. Regions of attenuated activity are shown in blue, whereas regions of amplified activity are shown in red.

The results of the frequency analysis by ICA for pParticipant C are shown in Figure 5.

The results of the ICA frequency analysis for Participant C indicated the following findings. In the PT informing condition, in the θ wave band, there was an amplification in activity in the left frontal pole and a reduction in activity in the left visual association area; in the α wave band, there was an amplification in activity in the right dorsolateral prefrontal cortex and a reduction in activity in the right visual association area; and in the β wave band, there was an amplification in activity in the right dorsolateral prefrontal cortex and a reduction in activity in the left somatosensory association area. In the mother informing condition, in the θ wave band, there was a reduction in activity in the somatosensory association area; in the α wave band, there was an amplification in activity in the bilateral orbitofrontal cortices; and in the β wave band, there was an amplification in activity in the ventral prefrontal cortex.

In the PT informing within mixed condition, in the θ wave band, there was an amplification in activity in the superior temporal gyrus and a reduction in activity in the somatosensory association areas; in the α wave band, there was a reduction in activity in the left frontal pole; and in the β wave band, there was an amplification in activity in the right somatosensory association area and a reduction in activity in the bilateral orbitofrontal cortices. In the mother informing within mixed condition, in the θ wave band, there was an amplification in activity in the right inferior temporal gyrus and a reduction in activity in the left visual association area; in the α wave band, there was an amplification in activity in the bilateral frontal poles and a reduction in activity in the right visual association area; and in the β wave band, there was an amplification in activity in the right frontal pole and a reduction in activity in the right angular gyrus.

In the PT asking condition, in the θ wave band, there was an amplification in activity in the bilateral somatosensory association areas and a reduction in activity in the left orbitofrontal cortex; in the α wave band, there was a reduction in activity in the orbitofrontal cortex; and in the β wave band, there was an amplification in activity in the somatosensory association areas and a reduction in activity in the middle temporal gyrus. In the mother asking condition, in the θ wave band, there was an amplification in activity in the right frontal pole; in the α wave band, there was an amplification in activity in the left somatosensory association area and a reduction in activity in the bilateral supplementary motor areas; and in the β wave band, there was an amplification in activity in the right dorsolateral prefrontal cortex.

In the PT asking within mixed condition, in the θ wave band, there was a reduction in activity in the bilateral orbitofrontal cortices; in the α wave band, there was a reduction in activity in the right frontal pole and the right somatosensory association area; and in the β wave band, there was an amplification in activity in the ventral prefrontal cortex. In the mother asking within mixed condition, in the θ wave band, there was an amplification in activity in the bilateral somatosensory association areas and a reduction in activity in the left frontal pole; in the α wave band, there was a reduction in activity in the bilateral frontal poles; and in the β wave band, there was an amplification in activity in the bilateral frontal eye fields and a reduction in activity in the left orbitofrontal cortex.

Summarizing the results for Participant C, under the PT informing condition, there was an increase in activity in the dorsolateral prefrontal cortex and a decrease in activity in the visual association cortex, suggesting that attention control was enhanced while visual information processing was suppressed. Under the mother informing condition, an increase in activity in the orbitofrontal cortex and ventromedial prefrontal cortex indicated that emotional processing was enhanced. Additionally, in the mother informing within mixed condition, an early increase in activity in the inferior temporal gyrus suggested that rapid language processing was taking place. In the PT informing condition, an increase in activity in the somatosensory association area and a decrease in activity in the orbitofrontal cortex indicated that attention was directed toward bodily sensations while emotional processing was suppressed. Under the mother informing condition, a decrease in activity in the supplementary motor area suggested that motor preparation was suppressed. In the PT informing within mixed condition, an increase in activity in the somatosensory association area and a decrease in activity in the orbitofrontal cortex indicated that sensory information processing was enhanced while behavioral inhibition was present. Under the mother informing within mixed condition, an increase in activity in the frontal pole and a decrease in activity in the angular gyrus suggested that decision-making was enhanced while responses to physical sensations were suppressed. Under the PT asking condition, an increase in activity in the somatosensory association areas and a decrease in activity in the middle temporal gyrus suggested that sensory information processing was enhanced while language processing was suppressed. Under the mother asking condition, a decrease in activity in the supplementary motor areas and an increase in activity in the dorsolateral prefrontal cortex indicated a reduction in the intention to move and an increase in emotional processing. In the PT asking within mixed condition, a decrease in activity in the somatosensory association areas and an increase in activity in the ventral prefrontal cortex indicated a reduction in sensory information processing and an increase in social behavior. In the mother asking within mixed condition, an amplification in activity in the frontal eye fields and a reduction in activity in the orbitofrontal cortex suggested an increase in emotional regulation and a decrease in social judgment.

## 4. Discussion

The ICA frequency analysis results for each participant revealed distinct brain activity patterns in response to verbal encouragement from the mother and PT, with variations observed across the θ (4–7 Hz), α (8–13 Hz), and β (14–30 Hz) frequency bands. These findings align with previous studies demonstrating the involvement of these frequency bands in cognitive processes such as attention, working memory, sensory processing, and motor control [17,18].

For Participant A, the PT informing condition showed an increase in β band activity in the somatosensory association cortex and a decrease in θ band activity in the inferior frontal gyrus, suggesting a focus on sensory information processing and a reduction in attention and working memory load. This aligns with prior research indicating the role of β oscillations in sensory processing and the inferior frontal gyrus in attention and working memory [19,20]. In the mother informing condition, α band activity increased in the frontal eye field, whereas β band activity decreased in the supplementary motor area and visual association area, indicating suppression of motor preparation and visual information processing in favour of auditory stimuli. These results are consistent with studies highlighting the role of alpha oscillations in auditory processing and the involvement of the frontal eye field, supplementary motor area, and visual association area in motor preparation and visual processing [21,22,23]. In the PT informing within mixed condition, activity increased in the right frontal pole within the θ wave band and decreased in the frontal eye fields within the β wave band, aligning with prior research on θ waves in decision-making and emotional processing and the prefrontal cortex in working memory [24,25,26]. Similarly, in the mother informing within mixed condition, α wave activity increased in the orbitofrontal cortex, whereas θ wave activity decreased in the somatosensory association areas, consistent with previous studies on α wave involvement in decision-making and emotional processing and sensory processing suppression in the somatosensory association areas [27,28]. For the PT asking condition, β wave activity increased in the frontal eye fields, whereas somatosensory association area activity decreased, supporting research on the orbitofrontal cortex’s role in goal-directed behavior and the suppression of sensory information processing in the somatosensory association areas [18,29]. In the mother asking condition, θ wave activity decreased in the orbitofrontal cortex, whereas β wave activity increased, which is consistent with previous findings on the orbitofrontal cortex’s role in attention tasks within the θ wave band and the somatosensory association areas’ involvement in concentration and understanding within the β wave band [18,30]. For the PT asking within mixed condition, α wave activity decreased in the orbitofrontal cortex, alongside a reduction in β wave activity in the somatosensory association areas, in line with studies on the orbitofrontal cortex’s role in internal cognitive processes and the β wave band’s association with concentration and understanding [18,31]. In the mother asking within mixed condition, α wave activity increased in the supplementary motor area, whereas β wave activity decreased in the orbitofrontal cortex, mirroring research on the supplementary motor area’s role in movement preparation and the orbitofrontal cortex’s function in emotional suppression [26,32].

These findings suggest that during the informing condition, Participant A exhibited increased attention and engaged in external cognitive activities that support cognitive function. Conversely, during the asking condition, the child may have focused on intrinsic cognitive processes, prioritizing internal cognitive engagement over external prompts.

Participant B exhibited distinct brain activity patterns in response to verbal encouragement across conditions. In the PT informing condition, θ band activity decreased in the dorsolateral prefrontal cortex, α band activity increased in the dorsal posterior cingulate cortex, and β band activity decreased in the somatosensory association cortex and supplementary motor area. This pattern suggests suppressed working memory and attention to external stimuli, enhanced sensory information processing and attentional focus, and reduced interest in bodily sensory input and motor intent. These results align with previous research on the dorsolateral prefrontal cortex’s role in working memory and attention [23], the dorsal posterior cingulate cortex’s involvement in sensory processing and attention [33], and the somatosensory association cortex and supplementary motor area’s role in sensory integration and motor planning [34]. In the mother informing condition, θ band activity increased in the left inferior frontal gyrus, β band activity decreased in the somatosensory association cortex, α band activity increased in the left visual association area, and θ band activity decreased in the left orbitofrontal cortex. This suggests enhanced working memory, reduced focus on bodily sensations, perceptual learning, and decreased attention to internal cognitive processes. These findings are supported by prior research on the inferior frontal gyrus in working memory [35], the somatosensory association cortex in body sensation processing [18], the visual association area in perceptual learning [36], and the orbitofrontal cortex’s role in attention and internal cognition [31]. In the PT informing within mixed condition, α band activity increased in the frontal pole, whereas β band activity decreased in the somatosensory association areas, consistent with the right frontal pole’s role in enhancing attention and the attenuation of somatosensory association areas in sensory information suppression [18,37]. In the mother informing within mixed condition, θ band activity decreased in the frontal pole, whereas β band activity increased in the frontal eye fields, aligning with studies linking θ wave reductions to decreased attention to cognitive tasks and the frontal eye fields to goal-directed behavior [29,38]. In the PT asking condition, α band activity increased in the somatosensory association areas, and β band activity increased in the frontal pole, aligning with findings on somatosensory association area activity in relaxation and the frontal pole’s role in cognitive enhancement [39,40]. In the mother asking condition, α band activity increased in the dorsolateral prefrontal cortex, whereas β band activity decreased in the frontal pole, consistent with research on the dorsolateral prefrontal cortex’s involvement in decision-making and the frontal pole’s role in suppressing higher cognitive processing [41,42]. In the PT asking within mixed condition, θ band activity decreased in the dorsolateral prefrontal cortex, whereas α band activity increased in the right frontal pole, supporting research on the dorsolateral prefrontal cortex’s role in suppressing working memory and external attention and the right frontal pole’s role in decision-making [23,40]. In the mother asking within mixed condition, θ band activity increased in the frontal pole, whereas β band activity increased in the middle temporal gyri, aligning with studies on the frontal pole’s role in decision-making and emotional processing [24,25] and the middle temporal gyri’s function in language comprehension [43].

These findings suggest that during the informing condition, Participant B focused on sensory and cognitive processing, efficiently integrating information whereas suppressing external stimuli. In contrast, during the asking condition, emotional and cognitive processes appeared to be integrated, facilitating efficient and socially oriented information processing.

The results for Participant C showed that in the PT informing condition, there was an increase in β band activity in the left frontal pole and a decrease in α band activity in the left visual association area, an increase in θ band activity in the right dorsolateral prefrontal cortex and a decrease in α band activity in the right visual association area, and an increase in β band activity in the right dorsolateral prefrontal cortex and a decrease in θ band activity in the left somatosensory association area. This pattern suggests an enhancement of goal-directed behavior, decision-making, and emotional processing, an inhibition of visual information processing, increased concentration, planning, and sustained attention, and a suppression of responses to sensory information. These results are consistent with previous research indicating the involvement of the frontal pole in goal-directed behavior and decision-making [23,25], the visual association area in visual processing [44], the dorsolateral prefrontal cortex in decision-making, concentration, planning, and sustained attention [45], and the somatosensory association area in sensory processing and inhibition [18]. In the mother informing condition, a decrease in θ band activity in the somatosensory association area, an increase in α band activity in the orbitofrontal cortex, and an increase in β band activity in the inferior frontal gyrus were observed, suggesting a reduction in attention to bodily sensations, enhanced internal cognitive processes, and increased empathy and social behavior. These findings align with studies showing the role of the somatosensory association area in body sensation processing [28,46], the orbitofrontal cortex in internal cognitive processes [31,47], and the ventromedial prefrontal cortex in empathy and social behavior [48]. In the PT informing within mixed condition, there was an amplification in activity in the superior temporal gyrus in the θ wave band and a decrease in activity in the orbitofrontal cortex in the β wave band, which aligns with prior research regarding the role of the superior temporal gyrus in language processing and the role of the orbitofrontal cortex in emotional processing [26,48]. Similarly, in the mother informing within mixed condition, there was an amplification in activity in the inferior temporal gyrus in the θ wave band and an increase in activity in the frontal pole in the α and β wave bands, findings that are consistent with previous studies highlighting the role of the inferior temporal gyrus in language processing and the role of the frontal pole in decision-making and cognitive function enhancement [40,49]. In the PT asking condition, a decrease in activity was observed in the orbitofrontal cortex in the θ wave band, whereas activity in the somatosensory association areas increased in the β wave band, and activity in the middle temporal gyrus decreased. This supports prior research indicating the role of the orbitofrontal cortex in enhancing concentration on tasks, the somatosensory association areas in attention to bodily sensations, and the inhibition of language and auditory processing by the middle temporal gyrus [18,30]. In the mother asking condition, an amplification in activity was noted in the frontal pole in the θ wave band and an increase in activity in the dorsolateral prefrontal cortex in the β wave band, which aligns with previous studies highlighting the role of the frontal pole in decision-making and emotional processing and the dorsolateral prefrontal cortex in emotional processing [24,25,26]. In the PT asking within mixed condition, there was a decrease in activity in the somatosensory association areas in the α wave band, whereas activity in the ventromedial prefrontal cortex increased in the β wave band. This aligns with prior research indicating the role of the somatosensory association areas in the inhibition of sensory processing and the ventromedial prefrontal cortex in empathy and social behavior [23,50]. Additionally, in the mother asking within mixed condition, there was a decrease in activity in the frontal pole in the α wave band, while activity in the frontal eye fields increased in the β wave band, which aligns with previous studies highlighting the involvement of the frontal pole in higher-order brain functions and planning and the role of the frontal eye fields in emotional regulation [27,51].

These findings suggest that although Participant C’s attention was directed toward auditory stimuli across all conditions, the child inhibited emotional evaluations and enhanced cognitive functions to engage in language processing during the PT asking and informing conditions, as well as the mother informing condition. In contrast, during the mother asking condition, the child may have engaged in emotional evaluations and focused attention on the social connection with the mother. Additionally, although attention was directed toward language processing during the PT’s inquiries and instructions and the mother’s instructions in the mixed condition, it is suggested that during the mother asking within mixed condition, the child focused on specific responses to the prompts.

The cognitive processes engaged by the participants differed not only depending on whether they were listening to instructions from the PT or the mother but also across different frequency bands. These frequency-specific effects align with previous research suggesting that different frequency bands are associated with distinct cognitive functions [52,53]. The theta band appears to be involved in attention, working memory, and internal cognitive processes [54,55], whereas the alpha band is associated with sensory processing, perceptual learning, and social behavior [56,57]. However, the beta band, seems to be involved in sensory processing, motor preparation, and goal-directed behavior [58,59].

The complex interplay between different frequency bands in mediating the cognitive processes engaged during verbal encouragement underscores the importance of considering these frequency-specific effects when designing interventions for children with CP-SMID. Previous studies have demonstrated that tailored interventions targeting specific frequency bands can enhance cognitive functions in various populations [60]. By selectively modulating the frequency bands associated with the desired cognitive processes, it may be possible to optimize the effectiveness of rehabilitation interventions for children with CP-SMID. However, it is important to acknowledge some limitations of the current study. The small sample size restricts the generalizability of the findings, and potential confounding factors such as sleep conditions, diet, relationship with the child, and environmental variables at the time of measurement were not sufficiently controlled [61,62]. Additionally, the observed individual differences in the results of this study can be attributed to the lack of control for age and cognitive function level. There are no appropriate assessment scales to evaluate cognitive function levels in children with severe multiple disabilities due to cerebral palsy, making it difficult to conduct such evaluations. Future studies should aim to replicate these results in larger samples while accounting for these variables as rigorously as possible, including age as a controlling factor. Additionally, the absence of coherence and time-frequency analyses in the present study limits a more comprehensive understanding of the functional connectivity between brain regions and the temporal dynamics of brain activity [63,64]. Incorporating these advanced analytical techniques in future research could yield valuable insights into the neural mechanisms underlying the effects of verbal encouragement on cognitive processing in children with CP-SMID.

Despite these limitations, the present study provides novel evidence of the differential effects of verbal encouragement from mothers and PTs on cognitive processing in children with CP-SMID, as well as the frequency-specific nature of these effects. Emotions serve as the foundation of motivation; therefore, changes in emotional states resulting from verbal prompts may influence children’s motivation. Additionally, the impact of these prompts on cognitive functions could lead to a greater understanding of tasks and related concepts among children. Thus, it can be concluded that these findings hold significant implications for optimizing rehabilitation interventions and fostering cognitive development in this population. By tailoring verbal encouragement to target specific frequency bands and cognitive processes, it may be possible to enhance the efficacy of these interventions and support cognitive development more effectively. Future research should build upon these findings by examining the effects of verbal encouragement in larger samples, across different developmental stages and contexts, and by employing more advanced analytical techniques to capture the intricate neural dynamics underlying these effects comprehensively.

## 5. Conclusions

This study provides key insights into the neural mechanisms underlying the effects of verbal encouragement on cognitive processing in children with CP-SMID. The findings indicate that cognitive engagement in these children varies based on the individual providing encouragement, their tone of voice, and the associated frequency bands of brain activity. Specifically, maternal encouragement appears to facilitate internal information processing and promote behavioral engagement, whereas therapist-led encouragement may play a role in emotion regulation. The findings indicate that verbal encouragement stimulates the emotions that are associated with changes in motivation and enhances cognitive activity. Thus, verbal encouragement plays a significant role in improving motivation, suggesting its potential to enhance the effectiveness of rehabilitation interventions. Furthermore, theta band activity is implicated in attention, working memory, and internal cognitive processes; alpha band activity is associated with sensory processing, perceptual learning, and social behavior; and beta band activity is linked to sensory processing, motor preparation, and goal-directed behavior.

These findings have significant implications for optimizing rehabilitation interventions and fostering cognitive development in children with CP-SMID. Tailoring verbal encouragement to target specific frequency bands and cognitive processes—while accounting for the individual providing encouragement and their tone of voice—could enhance the efficacy of these interventions. Furthermore, we are confident that this study can provide more effective interactions not only in rehabilitation settings but also in everyday life and therapeutic contexts to support children’s cognitive and emotional development. However, limitations such as the small sample size and potential confounding variables underscore the need for further research. Additionally, this study focused on whether the tone of verbal prompts is teaching or asking, encompassing a variety of content ranging from motivational to everyday topics. Therefore, it is necessary to further examine the content of verbal prompts in the future and to advance research based on both the content and tone that are effective for motivation.

Future studies should aim to replicate these findings in larger samples while rigorously controlling for confounding factors. Moreover, future studies should aim to replicate these findings in larger samples with more homogeneous age groups or by controlling for age as a variable to better understand the influence of age on the observed neural activity patterns. Additionally, incorporating coherence and time-frequency analyses could offer deeper insights into the functional connectivity between brain regions and the temporal dynamics of neural responses to verbal encouragement. The application of advanced neuroimaging techniques, such as functional near-infrared spectroscopy (fNIRS) [65,66], could further elucidate the underlying neural mechanisms.

Beyond methodological improvements, future research should explore the effects of verbal encouragement across different developmental stages and contexts. Investigating these mechanisms in typically developing children and those with other neurodevelopmental disorders may help identify shared and distinct neural activity patterns, guiding the development of tailored interventions. Moreover, examining verbal encouragement in varied settings, such as classrooms or social interactions, could provide valuable insights into the generalizability of these findings. Addressing these research gaps will contribute to evidence-based interventions that support cognitive development and overall well-being in children with CP-SMID.

## Figures and Tables

**Table 1:** Participant characteristics.

Participant Characteristics			
	A	B	C
Age (years)	3	7	9
Gender (boy/girl)	boy	girl	girl
Disease	cerebral palsy (CP)		
Classified	severe psychosomatic disorder		
Verbal Communication	vocalizing but struggling to produce meaningful words		
Motor Level	difficulty turning over; unable to move voluntarily	difficulty turning over; unable to move voluntarily	able to crawl a few meters

**Table 2:** Summary of the main brain activity areas in each frequency band under each condition.

Participant	Condition		Activity (Power Value)	θ-Wave	α-Wave	β-Wave
A	PT’s	informing condition	amplification (max)	somatosensory association areas (1.64 × 10^0^)	somatosensory association areas (1.54 × 10^0^)	right superior temporal gyrus (1.27 × 10^0^)
			reduction (minimum)	right inferior frontal gyrus (−1.75 × 10^0^)	visual association area (−1.45 × 10^0^)	
		asking condition	amplification (max)	frontal eye fields (1.68 × 10^0^)	right visual association area (7.79 × 10^0^)	frontal eye fields (1.02 × 10^0^)
			reduction (minimum)	somatosensory association areas (1.67 × 10^0^)	frontal poles (−8.78 × 10^0^)	somatosensory association areas (−1.40 × 10^0^)
		informing within mixed condition	amplification (max)	right frontal pole (8.63 × 10^−1^)	right visual association area (7.26 × 10^−1^)	somatosensory association areas (1.33 × 10^0^)
			reduction (minimum)	somatosensory association areas (−8.54 × 10^−1^)	supplementary motor areas (−6.7 × 10^−1^)	frontal eye fields (−1.29 × 10^0^)
		asking within mixed condition	amplification (max)	supplementary motor areas (1.32 × 10^0^)	supplementary motor areas (1.06 × 10^0^)	
			reduction (minimum)	orbitofrontal cortex (−1.39 × 10^0^)	orbitofrontal cortex (−1.03 × 10^0^)	somatosensory association areas (−1.54 × 10^0^)
	mother’s	informing condition	amplification (max)	frontal eye fields (7.76 × 10^−1^)	somatosensory association areas (7.78 × 10^−1^)	somatosensory association areas (−1.32 × 10^0^)
			reduction (minimum)	visual association areas (−9.43 × 10^0^)	supplementary motor areas (−7.93 × 10^0^)	left frontal pole (−1.46 × 10^0^)
		asking condition	amplification (max)	somatosensory association areas (1.08 × 10^0^)	visual association areas (1.15 × 10^0^)	somatosensory association areas (1.45 × 10^0^)
			reduction (minimum)	frontal eye fields (−1.38 × 10^0^)	frontal eye fields (−9.59 × 10^0^)	Inferior temporal gyrus (−1.40 × 10^0^)
		informing within mixed condition	amplification (max)	right somatosensory association area (1.33 × 10^0^)	right visual association area (1.51 × 10^0^)	supplementary motor areas (1.66 × 10^0^)
			reduction (minimum)	right dorsolateral prefrontal cortex (−1.50 × 10^0^)	left orbitofrontal cortex (−8.17 × 10^0^)	right orbitofrontal cortex (−1.36 × 10^0^)
		asking within mixed condition	amplification (max)	supplementary motor areas (2.10 × 10^0^)	supplementary motor areas (1.40 × 10^0^)	
			reduction (minimum)		frontal poles (−1.25 × 10^0^)	orbitofrontal cortex (−1.98 × 10^0^)
B	PT’s	informing condition	amplification (max)		dorsal posterior cingulate cortex (2.1 × 10^0^)	
			reduction (minimum)	dorsolateral prefrontal cortex (−3.64 × 10^0^)	supplementary motor areas (−2.46 × 10^0^)	frontal poles (−3.13 × 10^0^)
		asking condition	amplification (max)	orbitofrontal cortices (2.01 × 10^0^)	somatosensory association areas (1.56 × 10^0^)	frontal poles (1.65 × 10^0^)
			reduction (minimum)	supplementary motor areas (−1.93 × 10^0^)	supplementary motor areas (−1.69 × 10^0^)	supplementary motor areas (−1.57 × 10^0^)
		informing within mixed condition	amplification (max)	somatosensory association (2.18 × 10^0^)	right frontal pole (1.36 × 10^0^)	frontal poles (3.16 × 10^0^)
			reduction (minimum)	orbitofrontal cortex (−2.67 × 10^0^)	visual association areas (−2.45 × 10^0^)	somatosensory association areas (−3.30 × 10^0^)
		asking within mixed condition	amplification (max)	frontal pole (8.07 × 10^−1^)	right frontal pole (2.09 × 10^0^)	somatosensory association areas (1.26 × 10^0^)
			reduction (minimum)	dorsolateral prefrontal cortex (−8.12 × 10^−1^)		frontal pole (−2.39 × 10^0^)
	mother’s	informing condition	amplification (max)	left inferior frontal gyrus (1.88 × 10^0^)	left visual association area (1.25 × 10^0^)	right middle temporal gyrus (1.97 × 10^0^)
			reduction (minimum)	somatosensory association areas (−2.73 × 10^0^)	left orbitofrontal cortex (−1.42 × 10^0^)	left somatosensory association area (−2.53 × 10^0^)
		asking condition	amplification (max)	right middle temporal gyrus (2.29 × 10^0^)	right dorsolateral prefrontal cortex (1.52 × 10^0^)	left somatosensory association area (2.58 × 10^0^)
			reduction (minimum)		right visual association areas (−1.32 × 10^0^)	left frontal pole (−2.82 × 10^0^)
		informing within mixed condition	amplification (max)			left frontal eye fields (2.16 × 10^0^)
			reduction (minimum)	left frontal pole (−4.23 × 10^0^) right fusiform gyrus (−3.97 × 10^0^)	visual association areas (−2.22 × 10^0^)	visual association areas (−3.04 × 10^0^)
		asking within mixed condition	amplification (max)	left frontal pole (1.21 × 10^0^)	somatosensory areas (2.98 × 10^0^)	middle temporal gyri (1.66 × 10^0^)
			reduction (minimum)		frontal poles (−2.50 × 10^0^)	right frontal pole (−1.89 × 10^0^)
C	PT’s	informing condition	amplification (max)	left frontal pole (2.13 × 10^0^)	right dorsolateral prefrontal cortex (1.79 × 10^0^)	right dorsolateral prefrontal cortex (1.28 × 10^0^)
			reduction (minimum)	left visual association area (−2.11 × 10^0^)	right visual association area (−1.42 × 10^0^)	left somatosensory association area (−1.25 × 10^0^)
		asking condition	amplification (max)	somatosensory association areas (1.37 × 10^0^)		somatosensory association areas (1.33 × 10^0^)
			reduction (minimum)	left orbitofrontal cortex(−1.41 × 10^0^)	orbitofrontal cortex (−2.52 × 10^0^)	middle temporal gyrus (−1.38 × 10^0^)
		informing within mixed condition	amplification (max)	superior temporal gyrus (7.87 × 10^−1^)		right somatosensory association area (1.35 × 10^0^)
			reduction (minimum)	somatosensory association areas (−7.29 × 10^−1^)	left frontal pole (−2.36 × 10^0^)	orbitofrontal cortices (−2.01 × 10^0^)
		asking within mixed condition	amplification (max)			ventral prefrontal cortex (1.84)
			reduction (minimum)	orbitofrontal cortices (−1.1 × 10^0^)	right frontal pole (−9.80 × 10^−1^)	
	mother’s	informing condition	amplification (max)		orbitofrontal cortices (1.91 × 10^0^)	ventral prefrontal cortex (1.32 × 10^0^)
			reduction (minimum)	somatosensory association area (−1.88 × 10^0^)		
		asking condition	amplification (max)	right frontal pole (1.71 × 10^0^)	left somatosensory association area (8.68 × 10^−1^)	right dorsolateral prefrontal cortex (1.29 × 10^0^)
			reduction (minimum)		supplementary motor areas (−1.01 × 10^0^)	
		informing within mixed condition	amplification (max)	right inferior temporal gyrus (5.82 × 10^−1^)	frontal poles (1.40 × 10^0^)	right frontal pole (8.96 × 10^−1^)
			reduction (minimum)	left visual association area (−6.37 × 10^−1^)	right visual association area (−1.04 × 10^0^)	right angular gyrus (−1.55 × 10^0^)
		asking within mixed condition	amplification (max)	somatosensory association area (1.27 × 10^0^)		frontal eye fields (1.44 × 10^0^)
			reduction (minimum)	left frontal pole (−1.16 × 10^0^)	frontal poles (−7.69 × 10^−1^)	left orbitofrontal cortex (−1.55 × 10^0^)

## Data Availability

We are unable to share these research data. We do not have consent from the research participants, and the data contain personal information that we cannot share without authorization. It is necessary for us to respect the privacy of the research participants, and sharing their personal information without permission would not be appropriate. We appreciate your understanding.
